# A new tumor biomarker, serum protein peak at 3,144 *m*/*z*, in patients with node-positive breast cancer

**DOI:** 10.1007/s12094-014-1264-9

**Published:** 2014-12-16

**Authors:** Z. Chen, S. Xu, D. Su, W. Liu, H. Yang, S. Xie, X. Meng, L. Lei, X. Wang

**Affiliations:** 1 Department of Medical Oncology, Zhejiang Cancer Hospital, Hangzhou, Zhejiang 310022 China; 2 Key Laboratory Diagnosis and Treatment Technology on Thoracic Oncology, Zhejiang Cancer Institute, Zhejiang Province, Hangzhou, Zhejiang 310022 China; 3 Department of Breast Surgery, Zhejiang Cancer Hospital, Hangzhou, Zhejiang 310022 China

**Keywords:** Breast cancer, Serum, SELDI–TOF MS, 3,144 *m*/*z* protein peak, CA15-3

## Abstract

**Purpose:**

To explore the association between the 3,144 *m*/*z* protein peak and the clinicopathological features and prognosis in breast cancer.

**Methods:**

Using SELDI–TOF MS, we analyzed serum protein peak at 3,144 *m*/*z* in 283 patients with node-positive breast cancer, its relationship with clinicopathological features and their prognosis evaluating value of survival.

**Results:**

3,144 *m*/*z* positive rate was higher in elderly patients (42.8 % in ≥50-year-old vs. 31.2 % in <50, *P* = 0.04). However, no correlation was observed between 3,144 *m*/*z* and other clinicopathological features (body mass index, menstrual status, family history, TNM, molecular subtypes, vascular invasion, neural invasion, p53 and CA15-3). However, the positive rate of 3,144 *m*/*z* was higher than that of CA15-3 (35.5 vs. 11.4 %, McNemar *χ* ^2^ test, *p* < 0.001). 3,144 *m*/*z*-negative patients (*n* = 177) had a better 3-year overall survival (OS) than 3,144 *m*/*z*-positive patients (*n* = 106) (89.8 vs. 81.2 %, *P* = 0.045). Younger patients (*P* = 0.016), postmenopausal status (*P* = 0.019), small tumor (*P* < 0.001), less positive nodes (*P* < 0.001), early stage (*P* < 0.001), favorable molecular subtype (*P* = 0.007), normal CA15-3 (*P* = 0.003) and neoadjuvant chemotherapy (*P* = 0.001) predicted better survival. Cox analysis showed that T3–4 (95 % CI 1.419–8.057, *P* = 0.006), lymph node metastasis (95 % CI 1.242–3.632, *P* = 0.006) and p53 mutation (95 % CI 1.088–6.378, *P* = 0.032) were independent adverse prognostic factors. But childbirth ≥2 (95 % CI 0.163–0.986, *P* = 0.046), adjuvant chemotherapy (95 % CI 0.062–0.921, *P* = 0.038) and adjuvant radiotherapy (95 % CI 0.148–0.928, *P* = 0.034) were the independent factors in reducing risk of death in breast cancer patients. Combination testing of 3,144 *m*/*z* and CA15-3 will improve the prognosis value of 3-year survival (*P* = 0.011); patients with CA153−/3144− were characterized by the longest survival (89.8 %) and the CA153+/3144+ patients by the shortest.

**Conclusions:**

Serum protein peak at 3,144 *m*/*z* is a new biomarker for breast cancer diagnosis and prognosis and showed a higher positive rate than serum CA15-3. Combining 3,144 *m*/*z* and CA15-3 testing may improve prognosis of longer survival in breast cancer patients.

## Introduction

Breast cancer is one of the most common cancers in women. Despite a good long-term overall survival, recurrence and metastasis are primarily responsible for treatment failure [1]. Metastasis to the axillary lymph nodes is a key indicator of prognosis in breast cancer. The overall 5-year survival of breast cancer patients with positive axillary lymph node is lower than that of patients with negative lymph nodes, and there is almost a linear relationship between nodal disease burden and breast cancer-specific survival independent of tumor size. Recently, breast cancer serum tumor markers for early diagnosis, prognosis and recurrence monitoring have received increasing attention [2, 3]. CA15-3 is a commonly used marker in breast cancer management, and provides assistance for advanced breast cancer diagnosis and monitoring postoperative patients. However, the sensitivity of CA15-3 for diagnosis of advanced breast cancer is only 15.3 [4] to 22.5 % [5]. Thus, there is an urgent need for a simple, sensitive method for monitoring metastasis and recurrence in breast cancer [6].

The human proteome reflects all proteins and peptides which may be related to one gene and allows a more detailed evaluation of disease status. At present, it has become relatively easy to detect protein profiling in crude biological samples with surface-enhanced laser desorption/ionization time of flight mass spectrometry (SELDI–TOF MS). Surface-enhanced laser desorption ionization time-of-flight (SELDI–TOF) mass spectrometry (MS) is an innovative approach in proteomics and has been used in the clinical setting to study tumor protein biomarkers [7], seek new markers for early diagnosis and prognosis in breast cancer [8, 9], and identify a more sensitive marker for neoadjuvant chemotherapy in breast cancer [10]. However, only a few studies have attempted to identify new markers for metastasis [11, 12], and few analyses have been performed to study the relation between these markers and breast cancer survival. We previously used SELDI–TOF MS to analyze cell culture media and cell lysate from both high- and low-metastatic human ovarian cancer cell lines, and the results showed a differentially expressed protein peak at 3,144 *m*/*z* between these cells. These findings were preliminarily validated in serum samples from patients with advanced ovarian cancer [13].

Searching the Swiss protein database using the TagIdent online tool showed that a protein matched the 3,144 *m*/*z* peak and that it probably was CD24, which is a glycosyl phosphatidylinositol-anchored protein with mucin-like adhesion. Lee et al. [14] reviewed CD24 expression assessed by immunohistochemistry in 2,925 patients with cancer from 28 research reports. They observed an elevated expression of CD24 protein in a variety of cancers, including ovarian, breast, bladder, gastrointestinal, endometrial, bile duct, pancreatic, prostate and skin. CD24 may be involved in tumor development through the promotion of tumor cell proliferation, invasion and metastatic spread. It has become a biomarker and prognostic indicator for invasion and metastasis of certain malignant tumors [14]. Tissue CD24 expression levels may help to predict survival in patients with breast cancer [15], but studies show that highly invasive breast cancer cells often express CD44+/CD24− [16]. Such cells are considered to be breast cancer stem cells [17, 18].

So far, 3,144 *m*/*z* (CD24) expression in serum samples from breast cancer patients with positive lymph nodes has not been reported. This study aimed to analyze the protein peak at 3,144 *m*/*z* in serum samples from breast cancer patients with positive lymph nodes to determine the association between this protein peak and breast cancer prognosis, to evaluate its clinical implications and guide future research.

## Methods

### Patients’ characteristics

We retrospectively studied breast cancer patients admitted to the Zhejiang Cancer Hospital from August 2006 to June 2009. Patients were enrolled if they had breast cancer with positive lymph nodes. Diagnoses were established using surgical biopsy specimens. Clinical classification was made according to the Union for International Cancer Control (UICC) staging system (2010 edition). TNM was defined as: T1, tumor size ≤2 cm; T2, tumor size >2 and ≤5 cm; T3, tumor size >5 cm; T4, regardless of tumor size, a direct invasion into the chest wall (a) or skin (b); T4c = T4a + T4b; T4d, inflammatory breast cancer; N1, ipsilateral 1–3 lymph node positive; N2, ipsilateral 4–9 lymph node positive; N3: ipsilateral ≥10 lymph node positive or ipsilateral supraclavicular lymph node metastasis; and M1, distant metastasis. Patients with metastatic breast cancer and axillary lymph node metastasis from other primary tumors were excluded. General demographic data, pathological subtype, disease duration, and data on preoperative and postoperative treatments were collected. The study was approved by the ethics committee of Zhejiang Cancer Hospital and informed consent was obtained from all patients.

A total of 283 women with node-positive breast cancer with invasive ductal carcinoma after mastectomy were enrolled. There were 115 premenopausal and 164 postmenopausal women (4 patients had missing data) aged from 25 to 75 years (median 49.0 years). Diagnoses for all patients were confirmed by postoperative pathological examination. There were 117 cases at stage II and 166 cases at stage III–IV.

The tumor molecular subtypes were: luminal A (ER+ or PR+, HER2−) in 134 patients, luminal B (ER+ or PR+, HER2+) in 39 patients, HER2 positive (ER−/PR−/HER2+) in 33 patients and triple-negative or “basal-like” subtype (ER−/PR−/HER2−) in 72 patients. Five patients had no immunohistochemistry record and the subtypes were then unknown.

### Laboratory instruments and reagents

We used a PBS IIc SELDI–TOF MS (Ciphergen Biosystems, Fremont, CA, USA). Weak cation exchange (WCX) nanobeads, binding buffer and eluent products were purchased from Saierdi Inc. (Beijing, China). Acetonitrile, trifluoroacetic acid, SPA (sinapinic acid), urea, DTT, CHAPS, Tris–HCl and pure H_2_O were purchased from Sigma (St Louis, MI, USA).

### Sample collection and testing

#### Sample collection and preparation

Before first treatment (surgery or neoadjuvant chemotherapy), fasting peripheral blood samples were obtained from all patients and immediately placed at 4 °C for 1–2 h. Serum was separated by centrifugation at 4,000 rpm, at 4 °C for 5 min, and subsequently centrifuged at 14,000 rpm, at 4 °C for 5 min, to remove residual cell debris. Serum was transferred on ice to a new centrifuge tube and stored at −80 °C. Before testing, serum samples were thawed on ice. Serum samples (10 μl) were pipetted in 1.5 ml microcentrifuge tubes with 20 μl of 9 M urea buffer (9 mol/l urea, 2 % CHAPS, 50 mmol/l Tris–HCl, 1 % DTT, pH 9.0). Diluted samples were allowed to reach room temperature for 10 min, and 360 μl of binding buffer was then added.

#### Measurement of protein peak at 3,144 *m*/*z*

The detailed procedure has been previously published [13]. Briefly, WCX nanobeads were transferred into PCR tubes placed in a magnetic processor and liquid was removed. Following addition of 100 μl binding buffer, the PCR tubes were placed for 5 min in a magnetic processor to remove liquid and the same procedure was repeated once. Diluted serum sample (100 μl) was added to each PCR tube containing nanobeads. After mixing and reaching room temperature for 15 min, the PCR tubes were placed in the magnetic processor for removing unbound sample. Binding buffer (100 μl) was added to each tube; tubes were mixed and let to react for 5 min. The PCR tubes were then placed in the magnetic processor to discard liquid. The eluent (10 μl) was added to each tube and tubes were placed in the magnetic processor. 5 μl of supernatant was transferred to another PCR tube amd 5 μl of saturated SPA solution (sinapinic acid in 50 % acetonitrile and 0.5 % trifluoroacetic acid) was added and mixed well. Then, 1 μl was spotted onto an Au chip and allowed to air dry.

Before the chip was read on the PBS IIc mass spectrometer, NP20 chip with all-in-one standard proteins was used for instrument calibration, ensuring that the error in molecular weight ranged less than 0.1 %. The parameters of chip reading instrument were: laser intensity = 175; detection sensitivity = 8; optimization range = 1,000–15,000; and the highest molecular weight = 50,000. Each point on the chip was collected 90 times. Data were collected using the Ciphergen Protein Chip 3.2.1 software. According to the ROC curve of the protein peak (3,144 *m*/*z*) obtained from a previous study of pre-III–IV stage ovarian cancer [7], when the boundary value was set at 1.15, the sensitivity and specificity were 65.4 and 91.4 %, respectively. Therefore, the current study in breast cancer defined expression values of <1.15 as negative and of >1.15 as positive.

#### CA15-3 testing standards

We tested the CA15-3 with the same fasting peripheral blood samples as above. The Roche cancer antigen 15-3 (CA15-3) method is a sandwich electrochemiluminescence immunoassay that employs a biotinylated monoclonal CA15-3-specific antibody and a monoclonal CA15-3-specific antibody (Roche CA15-3 reagent, Roche Diagnostic Corp). The normal reference value was 0–28 U/ml. CA15-3 value over 28 U/ml was considered to be positive.

#### Tumor subtypes

Tumor subtypes were determined according to ER, PR and HER2 using immunohistochemistry [19]. Four micrometers-thick sections of formalin-fixed, paraffin-embedded tissue block of the best representative slide for each case were prepared for immunostains. Estrogen receptor (ER—monoclonal rabbit 1D5 clone), progesterone receptor (PR—monoclonal mouse PR636 clone), HER2 (rabbit immunoglobulin Hercep Test) and p53 (monoclonal mouse DO-7 clone) were performed using FDA approved antibodies. ER, PR and p53 were positive when ≥10 %. HER2 was positive (amplified/expressed) when 3+ in >30 % cells by immunohistochemistry. Cases with Hercep Test 2+ score (equivocal) were further analyzed for HER2 gene amplification by FISH (fluorescence in situ hybridization) technique.

### Follow-up

Follow-up was carried out in the outpatients receiving postoperative treatment or by telephone interview. It was completed on June 30, 2011.

### Data analysis and statistics

Data were analyzed using SPSS 16.0 (SPSS Inc., Chicago, IL, USA). Continuous data were described by frequency and rate. Positive rates between the different clinical and pathological features were examined using *χ* ^2^ tests. The association between 3,144 *m*/*z* protein peak and p53 was analyzed using McNemar *χ* ^2^ tests. Various factors affecting survival were analyzed using the Kaplan–Meier method and log-rank test. Meaningful variables and treatment data from univariate analysis were introduced into a Cox regression model to establish the independent prognostic factors. A *P* value <0.05 was considered to be statistically significant.

## Results

### Serum protein peak at 3,144 *m*/*z* and clinical features

The 3,144 *m*/*z* positive rate in the ≤50-year-old group was 31.1 % (47/151) and 44.7 % (59/132) in >50-year-old (χ^2^ = 5.537, *P* = 0.019). However, positive 3,144 *m*/*z* was not correlated with patients’ body mass index, menopausal status, family history, TNM stage, tumor molecular subtypes, vascular invasion, neural invasion, p53 expression and CA15-3 (Table 1).

**Table 1:** Relationship between patients’ characteristics and 3,144 *m*/*z* protein expression

	Protein peak at 3,144 *m*/*z*				χ^2^	*P* value
	*N*	Negative	Positive	Positive rate (%)		
Age (years)						
≤50	151	104	47	31.1	5.537	0.019
>50	132	73	59	44.7		
Blood type						
O	96	58	38	39.6	2.914	0.405
A	97	64	33	34.0		
B	67	38	29	43.3		
AB	23	17	6	26.1		
BMI^a^						
<24	159	103	56	35.2	0.485	0.486
≥24	117	71	46	39.3		
Menstruation^a^ (*n* = 279)						
Premenopausal	115	66	49	42.6	3.087	0.079
Postmenopausal	164	111	53	32.3		
Family history^b^ (*n* = 278)						
Yes	65	45	20	30.8	1.281	0.258
No	213	131	82	38.5		
Abortion^a^ (*n* = 278)						
No	179	111	68	38.0	0.597	0.440
Yes	99	66	33	33.3		
Menarche^a^ (years) (*n* = 277)						
>15	120	79	41	34.2	0.343	0.558
≤15	157	98	59	37.6		
Childbirth^a^ (*n* = 278)						
≥2	141	89	52	36.9	0.037	0.847
<2	137	88	49	35.8		
Tumor size						
T1–T2	233	145	88	37.8	0.055	0.815
T3–T4	50	32	18	36.0		
Lymphovascular invasion						
No	150	94	56	37.3	0.002	0.964
Yes	133	83	50	37.6		
Neural invasion						
No	249	154	95	38.2	0.430	0.512
Yes	34	23	11	32.4		
Lymph node metastasis						
N1	130	83	47	36.2	1.086	0.581
N2	86	50	36	41.9		
N3	67	44	23	34.3		
Clinical staging						
Stage II	117	71	46	39.3	0.295	0.587
Stage III–IV	166	106	60	36.1		
Subtype^a^ (*n* = 278)						
Luminal A	134	87	47	35.1	0.911	0.823
Luminal B	39	25	14	35.9		
HER2 (+)	33	21	12	36.4		
Basal-like	72	42	30	41.7		
p53						
Negative	109	72	37	33.9	4.382	0.112
Positive	147	93	54	36.7		
Unknown	27	12	15	55.6		
CA15-3^a^ (*n* = 220)						
Negative	195	124	71	36.4	0.685	0.408
Positive	25	18	7	28.0		

*BMI* body mass index

^a^With missing data

^b^Any other family member with cancer

**Table 2:** Comparison of positive percentage between 3,144 *m*/*z* and CA15-3

	Protein peak at 3144 m/z		Total
	Negative	Positive	
CA15-3			
Negative	124 (63.6)	71 (36.4)	195
Positive	18 (72.0)	7 (28.0)	25
Total	142 (64.5)	78 (35.5)	220

McNemar χ^2^ test, *P* < 0.001

### Serum protein peak at 3,144 *m*/*z* and prognosis

**Fig. 1:**
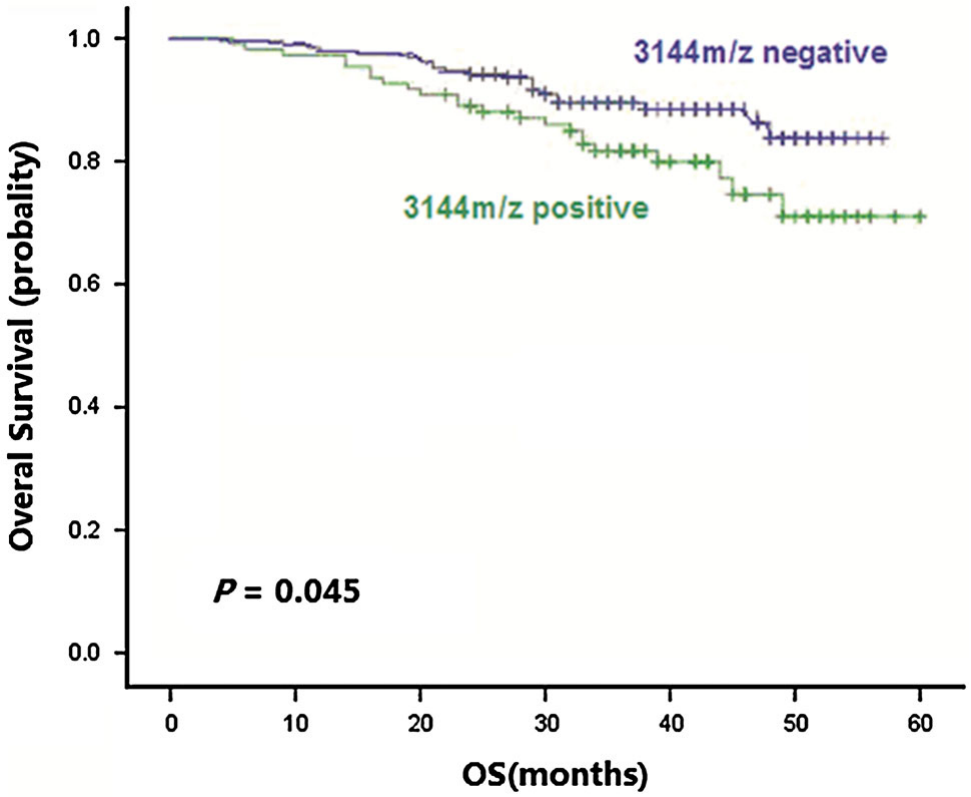
Kaplan–Meier estimates of overall survival for breast cancer with 3144 *m*/*z* protein positive and negative

Follow-up was completed on June 30, 2011. 244 of 283 patients with breast cancer survived and 39 patients died. The 3-year survival rate was 86.2 %. Kaplan–Meier survival analysis (Table 3) showed that the 3,144 *m*/*z* protein peak was related with overall survival in breast cancer patients. Positive protein expression at 3,144 *m*/*z* in 106 patients had a 3-year survival of 81.2 %, which was significantly lower than that in 177 patients with negative expression with a 3-year survival of 89.8 % (Log-Rank, *χ* ^2^ = 4.403, *P* = 0.045) (Fig. 1). In univariate analyses, the 3-year overall survival in breast cancer patients was associated with age, menopausal status, tumor size, lymph node metastasis, clinical stage, molecular typing, CA15-3, 3,144 *m*/*z* protein peak and neoadjuvant chemotherapy (all *P* < 0.05).

**Table 3:** Comparison of different breast cancer clinicopathological features and 3-year survival

Clinicopathological factors	*N*	3-year survival rate (%)	Log-rank *χ* ^2^	*P* value
Age (years)				
≤50	151	92.0	5.779	0.016
>50	132	79.8		
Blood type				
O	96	91.5	2.224	0.527
A	97	85.5		
B	67	79.9		
AB	23	97.0		
BMI				
<24	159	86.4	0.009	0.926
≥24	117	87.0		
Menstruation				
Premenopausal	115	79.5	5.478	0.019
Postmenopausal	164	91.3		
Family history^b^				
Yes	65	87.3	0.110	0.741
No	213	85.1		
Abortion				
No	179	84.2	1.980	0.159
Yes	99	90.3		
Menarche^a^ (years)				
>15	120	85.8	0.065	0.799
≤15	157	86.7		
Childbirth^a^				
≥2	141	88.6	2.075	0.150
<2	137	84.2		
Tumor size				
T1–T2	233	90.1	24.555	<0.001
T3–T4	50	67.6		
Intravascular cancer embolus				
No	150	86.6	0.398	0.528
Yes	133	85.8		
Neural invasion				
No	249	87.2	0.402	0.526
Yes	34	78.9		
Lymph node metastasis				
N1	130	94.3	16.435	<0.001
N2	86	86.6		
N3	67	68.2		
Clinical staging				
II	117	94.5	11.635	<0.001
III–IV	166	80.2		
Subtype^a^				
Luminal A	134	92.9	12.196	0.007
Luminal B	39	89.0		
HER2 (+)	33	86.7		
Basal-like	72	73.1		
p53				
Negative	109	90.5	2.754	0.097
Positive	147	81.8		
CA15-3^a^				
Negative	195	89.9	8.94	0.003
Positive	25	65.3		
Protein peak at 3,144 *m*/*z*				
Negative	177	89.8	4.403	0.045
Positive	106	81.2		
Neoadjuvant chemotherapy				
No	128	78.0	11.071	0.001
Yes	155	93.0		
Adjuvant chemotherapy				
No	14	78.6	1.593	0.207
Yes	269	86.8		
Adjuvant radiotherapy				
No	128	84.7	0.674	0.412
Yes	155	87.3		

*BMI* body mass index

^a^With missing data

^b^Any other family member with cancer

Cox regression analysis showed (Table 4) that T3–4, lymph node metastasis, p53 mutation, childbirth, adjuvant chemotherapy and radiotherapy were independent prognostic factors in this group of patients with breast cancer. The patients with T3–4 had 3.381-fold risk of death compared with T12 patients (95 % CI 1.419–8.057, *P* = 0.006); Cox proportional hazards model analysis showed that the patients with N3 lymph node metastasis had a 2.124-fold risk of death compared with patients with N1 + N2 lymph node metastasis (95 % CI 1.242–3.632, *P* = 0.006). However, childbirth ≥2, adjuvant chemotherapy and adjuvant radiotherapy were the independent factors in reducing the risk of death in breast cancer patients. 3,144 *m*/*z* expression was not an independent prognostic factor in patients with invasive ductal carcinoma in our study.

**Table 4:** COX multivariate analysis of prognostic factors in breast cancer patients

	*B*	SE	Wald	*df*	Sig	Exp(*B*)	95.0 % CI for Exp (*B*)	
							Lower	Upper
T-stage	1.218	0.443	7.563	1	0.006	3.381	1.419	8.057
LN status	0.753	0.274	7.578	1	0.006	2.124	1.242	3.632
Childbirth ≥2	−0.915	0.460	3.964	1	0.046	0.401	0.163	0.986
p53	0.969	0.451	4.610	1	0.032	2.634	1.088	6.378
Adjuvant CT	−1.427	0.686	4.325	1	0.038	0.240	0.062	0.921
Adjuvant RT	−0.992	0.468	4.494	1	0.034	0.371	0.148	0.928

*LN status* N1, N2 and N3, *CT* chemotherapy, *RT* radiotherapy

### Serum protein peak at 3,144 *m*/*z* and CA15-3

**Fig. 2:**
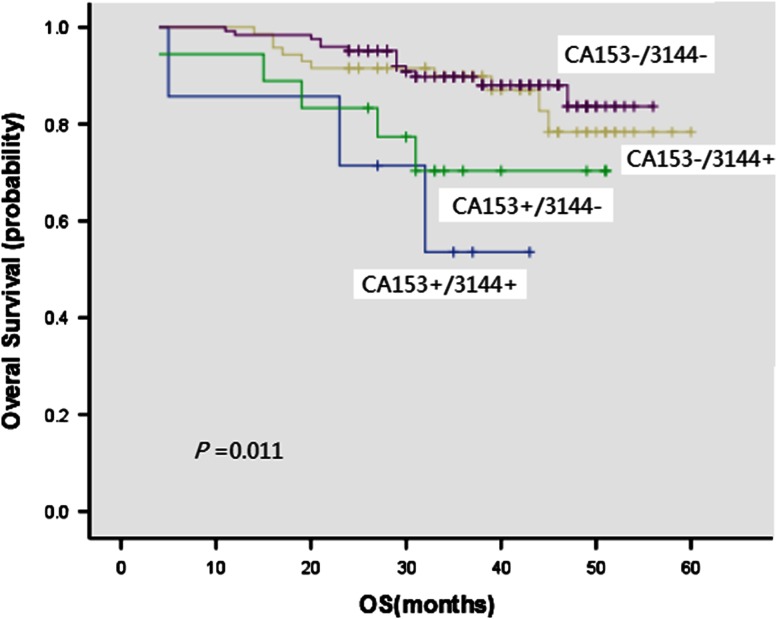
Kaplan–Meier estimates of overall survival for breast cancer with different CA153/3144 status

In 283 patients with breast cancer, 37.5 % (106/283) were preoperatively detected with a positive protein peak at 3,144 *m*/*z*. Of these patients, 220 patients were tested for CA15-3 and showed a positive result in 11.4 % (25/220). The difference between the two methods was statistically significant (McNemar χ^2^ test, *P* < 0.001), indicating that the 3,144 *m*/*z* protein pattern in breast cancer patients had a higher positive rate than the traditional CA15-3 marker. Combination testing of 3,144 *m*/*z* and CA15-3 will improve the prognosis value of 3-year survival (*P* = 0.011, Fig. 2). Patients with CA153−/3,144− were characterized by the longest survival (89.8 %) and CA153+/3,144+ patients by the shortest (53.6 %, Table 5).

**Table 5:** The different CA153/3,144 statuses and 3-year survival of breast cancer patients

Clinicopathological factors	*N*	3-year survival rate (%)	Log-rank *χ* ^2^	*P* value
CA153+/3,144+	7	53.6		
CA153+/3,144−	18	70.3	11.232	0.011
CA153−/3,144+	71	89.9		
CA153−/3,144−	124	89.8		

## Discussion

Over the past 30 years, the survival of women with early-stage breast cancer has been prolonged [20]. In addition to earlier detection, the use of adjuvant chemotherapy and endocrine therapy following definitive surgery and radiation therapy is credited with a significant improvement in overall survival. Traditionally, the prognosis of breast cancer has been known to be associated with tumor size, nodal status, hormonal receptor status, histologic grade, nuclear grade, human epidermal growth factor receptor 2 (HER2) expression, Ki-67 expression, etc. [21, 22]. However, positive axillary lymph node is a key indicator of prognosis in breast cancer patients. In recent years, cancer diagnostics has been taking enormous advantage of genomics and proteomics, novel fields of modern biology. Proteomics is the study of the proteome, which comprises the complete protein components of the cell, tissue or organism. The milestone paper, which was published in 2002 by the group of Petricoin and Liotta [23], showed that components of the serum proteome identified by mass spectrometry differentiated patients with ovarian cancer from healthy individuals. Compared to diagnostic studies, there were no satisfactory serum markers for early detection of the relapse of breast cancer after surgery and adjuvant therapy, and few reports were seen with SELDI–TOF MS being used in the prognosis for breast cancers with varying conclusions.

The molecular difference was researched by the gene microarray of breast cancer, and different gene expression profiles were found, which were classified into basal-like type, HER2 type, normal breast-like type and luminal-type. Luminal-type breast cancer was characterized by profiles of estrogen receptor (ER) positive or progesterone receptor (PR) positive; it showed better prognosis than other subtypes, such as basal-like type and HER2 type. In search of these markers, investigators from our institutes and hospital have published gene expression profiles in tumor tissue that outperformed all prognostic parameters in predicting disease outcome. One of the proteomic technologies used extensively in the search for novel markers is surface-enhanced laser desorption/ionization time-of-flight mass spectrometry (SELDI–TOF MS). The proteome might have greater ability in reflecting the molecular complexity of breast cancer. Postoperative serum protein pattern may provide prognostic information, since it reflects the host response to metastasis. The candidate prognostic marker found in the current study is most likely related to a postoperative host response. In addition, as patients were treated with adjuvant chemotherapy, a post-treatment pattern of differentially expressed proteins may represent tumor phenotype and chemosensitivity. These proteins produced during host response are generally present at substantially higher circulatory concentrations than the enzymes that process them upon their exposure to the tumor microenvironment, so they can be detected in the blood by SELDI–TOF MS.

This study used SELDI–TOF MS for detection of protein peak at 3,144 *m*/*z* in pretreatment peripheral blood samples from 283 patients with breast cancer and lymph node metastasis. There were no correlations between positive rate of protein peak at 3,144 *m*/*z* and CA15-3 in 220 patients (Table 2). The positive rate of the protein peak at 3,144 *m*/*z* (35.5 %) was significantly higher than that of CA15-3 (11.4 %). Although the traditional CA15-3 tumor marker has a low positive rate, the present study showed that CA15-3-positive patients had a lower survival rate, accompanied by other independent prognostic factors, thus defining a poor prognosis in these patients. Positive CA15-3 was mostly observed in patients at a late cancer stage. In fact, the prognostic value of CA15-3 in advanced breast cancer has already been appreciated [24]. However, there is a lack of a sensitive marker for patients with early breast cancer. To solve this problem, we conducted this study and observed that the positive rate of 3,144 *m*/*z* protein peak in lymph node metastasis and early stage breast cancer was significantly higher than CA15-3, and that 3-year survival rate of 107 patients with positive protein peak was significantly lower than the survival rate of 177 patients with negative expression. Despite that univariate analyses suggested a prognostic significance of 3,144 *m*/*z* protein peak, multivariate analyses did not confirm its independent prognostic value in breast cancer. But the combination testing of 3,144 *m*/*z* and CA15-3 will improve the prognosis value of 3-year survival (*P* = 0.011). The patients with CA153−/3,144− were characterized by the longest survival and the CA153+/3,144+ patients by the shortest.

The 3,144 *m*/*z* protein peak is a new biomarker for diagnosis and prognosis of breast cancer using peripheral blood, which is an ideal test specimen, easy to obtain and ready to be tested in various stages of the disease during follow-up. Nevertheless, we should note that a large proportion of the breast cancer patients with recurrence and metastasis had normal serum biomarkers levels, and that about 5 % of healthy people may have abnormal markers [13]. Besides, there are obvious limitations of searching proteins according to their molecular size, because some proteins share the same molecular weight. Future research should obtain sufficient data in highly metastatic human breast cancer cells through some method for further characterization of the protein at 3,144 *m*/*z*. The serum protein peak detected at 3,144 *m*/*z* combined with CA15-3 may provide a useful marker for diagnosis and prognosis of breast cancer.

Molecular subtypes proposed by Goldhirsch et al. [19] at the St Gallen International Breast Cancer Conference (2011) were widely adopted. In-depth study of molecular subtypes in breast cancer offers guidance on appropriate and effective treatment management for clinicians, thus avoiding inadequate treatment or overtreatment [25]. Cox proportional hazards model showed that neoadjuvant chemotherapy was an independent prognostic factor in breast cancer patients and demonstrated that surgery supplemented with chemotherapy and radiotherapy can increase survival. Cox proportional hazards model analysis showed that neoadjuvant chemotherapy prior to surgery was a prognostic factor, but did not reach statistical significance in the multivariate analysis; further in-depth study is needed to clarify such difference [26].

The current study suffered from some drawbacks. For example, chemotherapy, endocrine therapy and targeted therapy were all included in chemotherapy, but a stratified analysis will be performed in a future research. Also, a study including a large number of patients is required to confirm the prognostic significance of the 3,144 *m*/*z* protein peak.

In summary, the peripheral serum protein peak at 3,144 *m*/*z* provides an innovative, practical biomarker for diagnosis and prognosis of breast cancer. The simultaneous testing of serum CA15-3 may improve the detection rate of patients with breast cancer and lymph node metastasis.

## References

[1] de Boer M, van Deurzen CHM, van Dijck JAAM, Borm GF, van Diest PJ, Adang EMM (2009). Micrometastases or isolated tumor cells and the outcome of breast cancer. N Engl J Med.

[2] Russnes HG, Vollan HKM, Lingjærde OC, Krasnitz A, Lundin P, Naume B (2010). Genomic architecture characterizes tumor progression paths and fate in breast cancer patients. Sci Transl Med.

[3] Sotiriou C, Pusztai L (2009). Gene-expression signatures in breast cancer. N Engl J Med.

[4] Molina R, Augé J, Escudero J, Filella X, Zanon G, Pahisa J (2010). Evaluation of tumor markers (HER-2/neu oncoprotein, CEA, and CA15.3) in patients with locoregional breast cancer: prognostic value. Tumor Biol.

[5] Gion M, Mione R, Leon AE, Dittadi R (1999). Comparison of the diagnostic accuracy of CA27.29 and CA15.3 in primary breast cancer. Clin Chem.

[6] Musgrove EA, Sutherland RL (2009). Biological determinants of endocrine resistance in breast cancer. Nat Rev Cancer.

[7] Bakry R, Rainer M, Huck CW, Bonn GK (2011). Protein profiling for cancer biomarker discovery using matrix-assisted laser desorption/ionization time-of-flight mass spectrometry and infrared imaging: a review. Anal Chim Acta.

[8] Opstal-van Winden AJ, Vermeulen RH, Peeters PM, Beijnen J, Gils C (2011). Early diagnostic protein biomarkers for breast cancer: how far have we come?. Breast Cancer Res Treat.

[9] Böhm D, Keller K, Wehrwein N, Lebrecht A, Schmidt M, Kölbl H (2011). Serum proteome profiling of primary breast cancer indicates a specific biomarker profile. Oncol Rep.

[10] Zhang K, Yuan K, Wu H, Li Q, Wang Y, Chen S (2012). Identification of potential markers related to neoadjuvant chemotherapy sensitivity of breast cancer by SELDI–TOF MS. Appl Biochem Biotechnol.

[11] Wang L, Su D, Yan HJ, Xu JH, Zheng ZG, Hu YJ (2011). Primary study of lymph node metastasis-related serum biomarkers in breast cancer. Anat Rec: Adv Integr Anat Evolut Biol.

[12] Lei L, Wang XJ, Zheng ZG, Huang J, Cao WM, Chen ZH (2011). Identification of serum protein markers for breast cancer relapse with SELDI–TOF MS. Anat Rec: Adv Integr Anat Evolut Biol.

[13] Zheng Z, Gao Y, Gu L, Mou H, Zhu C, Zhu J (2009). Discovery of metastasis-associated biomarkers in ovarian cancer using SELDI–TOF: an in vitro and clinical study. Clin Oncol Cancer Res.

[14] Lee JH, Kim SH, Lee ES, Kim YS (2009). CD24 overexpression in cancer development and progression: a meta-analysis. Oncol Rep.

[15] Kristiansen G, Winzer KJ, Mayordomo E, Bellach J, Schlüns K, Denkert C (2003). CD24 expression is a new prognostic marker in breast cancer. Clin Cancer Res.

[16] Sheridan C, Kishimoto H, Fuchs R, Mehrotra S, Bhat-Nakshatri P, Turner C (2006). CD44 +/CD24− breast cancer cells exhibit enhanced invasive properties: an early step necessary for metastasis. Breast Cancer Res C7-R59.

[17] Leth-Larsen R, Terp MG, Christensen AG, Elias D, Kuhlwein T, Jensen ON (2012). Functional heterogeneity within the CD44 high human breast cancer stem cell-like compartment reveals a gene signature predictive of distant metastasis. Mol Med.

[18] Adamczyk A, Niemiec JA, Ambicka A, Mucha-Malecka A, Mitus J, Rys J. CD44/CD24 as potential prognostic markers in node-positive invasive ductal breast cancer patients treated with adjuvant chemotherapy. J Mol Histol. 2013 [Epub ahead of print]. doi:10.1007/s10735-013-9523-6. 10.1007/s10735-013-9523-6 23835592

[19] Goldhirsch A, Wood WC, Coates AS, Gelber RD, Thurlimann B, Senn HJ (2011). Strategies for subtypes—dealing with the diversity of breast cancer: highlights of the St Gallen International Expert Consensus on the primary therapy of early breast cancer 2011. Ann Oncol.

[20] Nishimura R, Osako T, Nishiyama Y, Tashima R, Nakano M, Fujisue M (2013). Evaluation of factors related to late recurrence—later than 10 years after the initial treatment—in primary breast cancer. Oncology.

[21] Jones T, Neboori H, Wu H, Yang Q, Haffty BG, Evans S (2013). Are breast cancer subtypes prognostic for nodal involvement and associated with clinicopathologic features at presentation in early-stage breast cancer?. Ann Surg Oncol.

[22] Lips EH, Mulder L, de Ronde JJ, Mandjes IA, Koolen BB, Wessels LF (2013). Breast cancer subtyping by immunohistochemistry and histological grade outperforms breast cancer intrinsic subtypes in predicting neoadjuvant chemotherapy response. Breast Cancer Res Treat.

[23] Petricoin EF, Ardekani AM, Hitt BA, Levine PJ, Fusaro VA, Steinberg SM (2002). Use of proteomic patterns in serum to identify ovarian cancer. Lancet.

[24] Younes A, Aggarwall BB (2003). Clinical implications of the tumor necrosis factor family in benign and malignant hematologic disorders. Cancer.

[25] Sørlie T, Perou CM, Tibshirani R, Aas T, Geisler S, Johnsen H (2001). Gene expression patterns of breast carcinomas distinguish tumor subclasses with clinical implications. Proc Natl Acad Sci.

[26] Parker JS, Mullins M, Cheang MCU, Leung S, Voduc D, Vickery T (2009). Supervised risk predictor of breast cancer based on intrinsic subtypes. J Clin Oncol.

